# Supplementary material for: 2D CdPS3-based versatile superionic conductors
Source: Nat Commun. 2023 Jul 6;14:3998. doi: 10.1038/s41467-023-39725-6 (PMC10326001; doi:10.1038/s41467-023-39725-6)
Supplement: Supplementary file 1 — Supplementary Information [file 41467_2023_39725_MOESM1_ESM.pdf]

Supplementary Information for

2D $CdPS_3$-based versatile superionic conductors

Yu *et al.*

Supplementary Figures

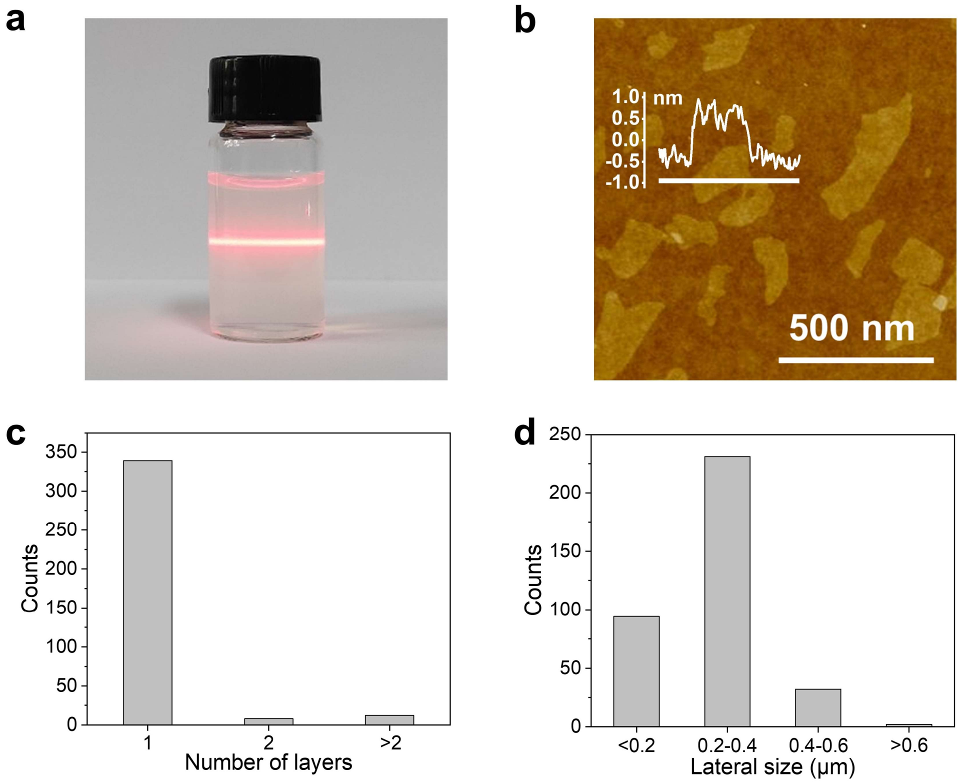

Supplementary Figure 1 | Characterizations of $Cd_{0.85}PS_3Li_{0.3}$ nanosheets. a, Photograph of $Cd_{0.85}PS_3Li_{0.3}$ nanosheet dispersion. **b**, Typical AFM image of $Cd_{0.85}PS_3Li_{0.3}$ nanosheets. Inset: height profile along the white line. **c**, **d**, Thickness (**c**) and lateral size distributions (**d**) of $Cd_{0.85}PS_3Li_{0.3}$ nanosheets.

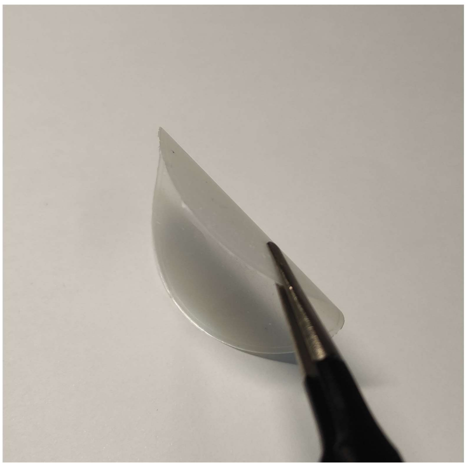

Supplementary Figure 2 | A photo of a free-standing $CdPS_3$-Li ($Cd_{0.85}PS_3Li_{0.3}$) membrane.

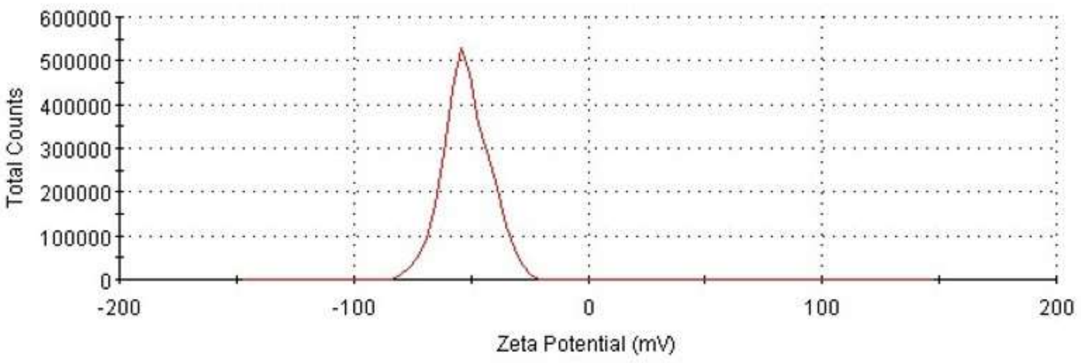

Supplementary Figure 3 | Zeta potential of $Cd_{0.85}PS_3Li_{0.3}$ nanosheet dispersion.

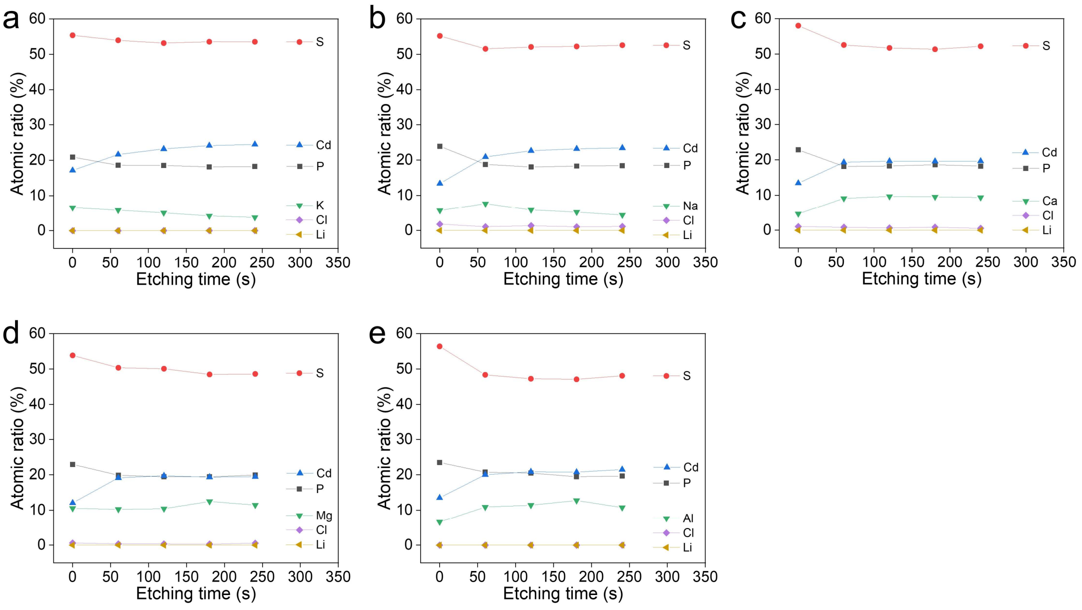

Supplementary Figure 4 | Characterization of exchanging Li^+ in the interlayer spacing to other cations. a-e, The quantified atomic ratio of elements in $CdPS_3$-Li membranes measured by XPS spectra with depth analysis after immersed in 0.5 M KCl (**a**), 0.5 M NaCl (**b**), 0.5 M $CaCl_2$ (**c**), 0.5 M $MgCl_2$ (**d**), and 0.5 M $AlCl_3$ (**e**) for over 48 h. These results suggest that Li^+ in the interlayer spacing was entirely replaced by the corresponding ions.

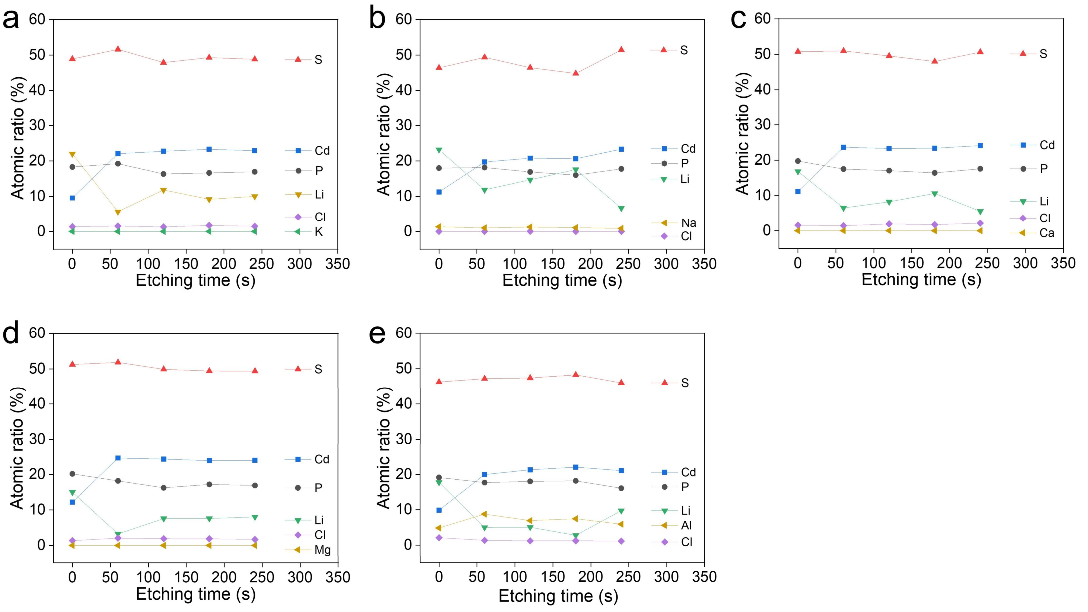

Supplementary Figure 5 | Characterization of re-exchanging Z cations in the interlayer spacing back to Li^{+}. a-e, The quantified atomic ratio of elements in $CdPS_3$-K (**a**), $CdPS_3$-Na (**b**), $CdPS_3$-Ca (**c**), $CdPS_3$-Mg (**d**), and $CdPS_3$-Al (**e**) membranes measured by XPS spectra with depth analysis after immersed in 0.5 M LiCl for over 48 h. These results show that K^{+}, Na^{+}, Ca^{2+} and Mg^{2+} in the interlayer spacing can be entirely replaced by Li^{+}, while Al^{3+} cannot be completely replaced.

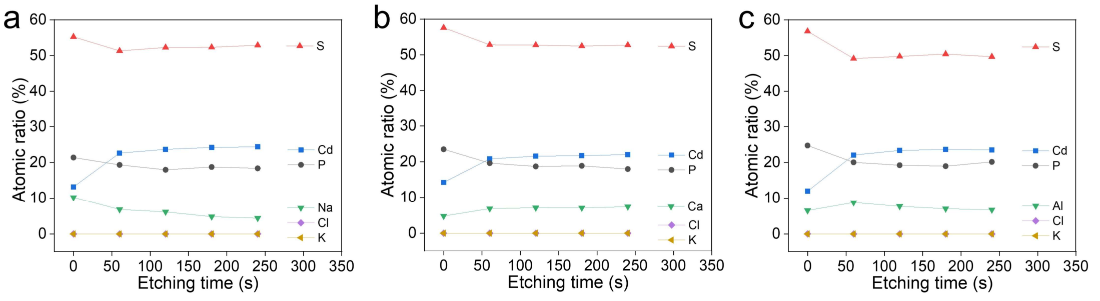

Supplementary Figure 6 | Characterization of exchanging K^{+} in the interlayer spacing to other cations. a-e, The quantified atomic ratio of elements in $CdPS_3$-K membranes measured by XPS spectra with depth analysis after immersed in 0.5 M NaCl (**a**), 0.5 M $CaCl_2$ (**b**), and 0.5 M $AlCl_3$ (**c**) for over 48 h. These results suggest that K^{+} in the interlayer spacing was entirely replaced by the corresponding ions.

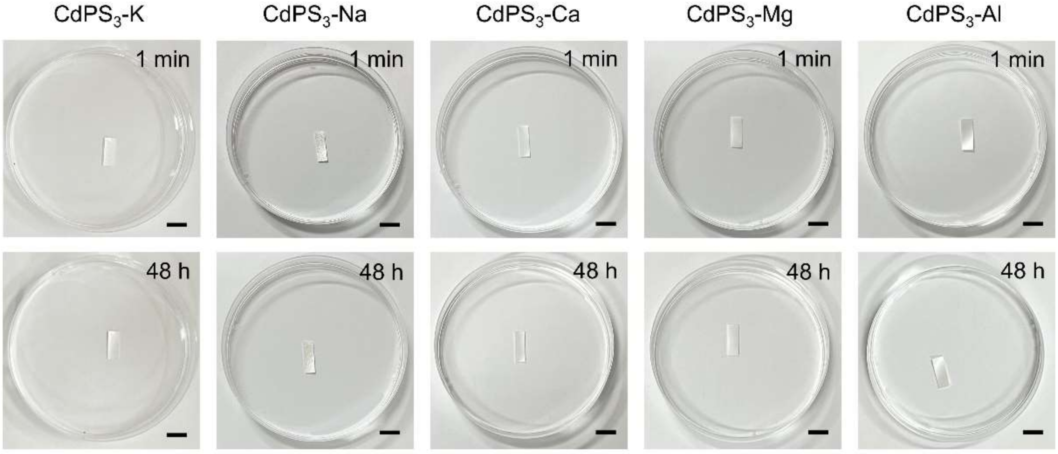

Supplementary Figure 7 | The stability of CdPS3-K, CdPS3-Na, CdPS3-Ca, CdPS3-Mg and CdPS3-Al membranes in water. Scale bar: 1 cm.

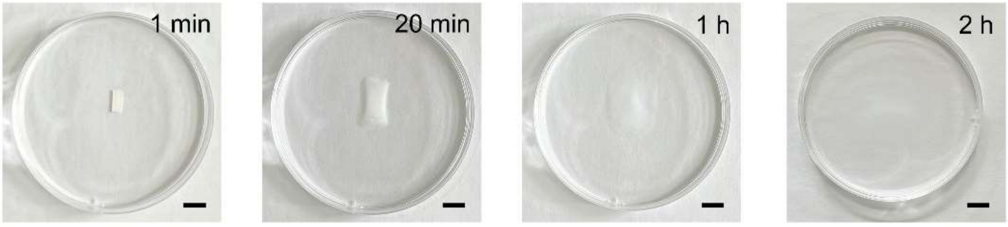

Supplementary Figure 8 | The stability of CdPS3-Li membrane in water. Scale bar: 1 cm.

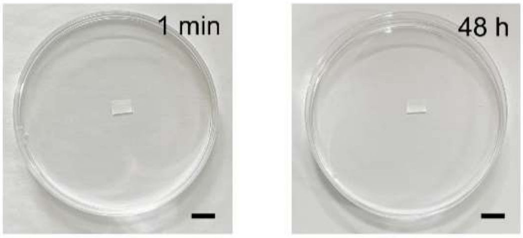

Supplementary Figure 9 | The stability of CdPS3-Li membrane in 0.5 M LiCl. Scale bar: 1 cm.

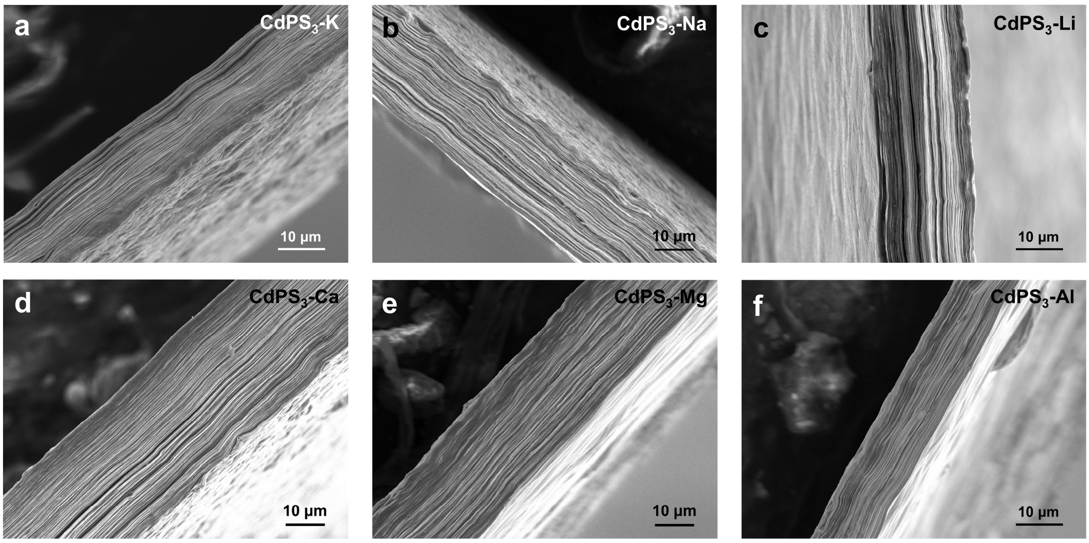

Supplementary Figure 10 | Structure characterizations of $CdPS_3$-Z membranes. **a-f**, Cross-sectional SEM images of $CdPS_3$-K (**a**), $CdPS_3$-Na (**b**), $CdPS_3$-Li (**c**), $CdPS_3$-Ca (**d**), $CdPS_3$-Mg (**e**), and $CdPS_3$-Al membranes (**f**), showing well-ordered lamellar structures.

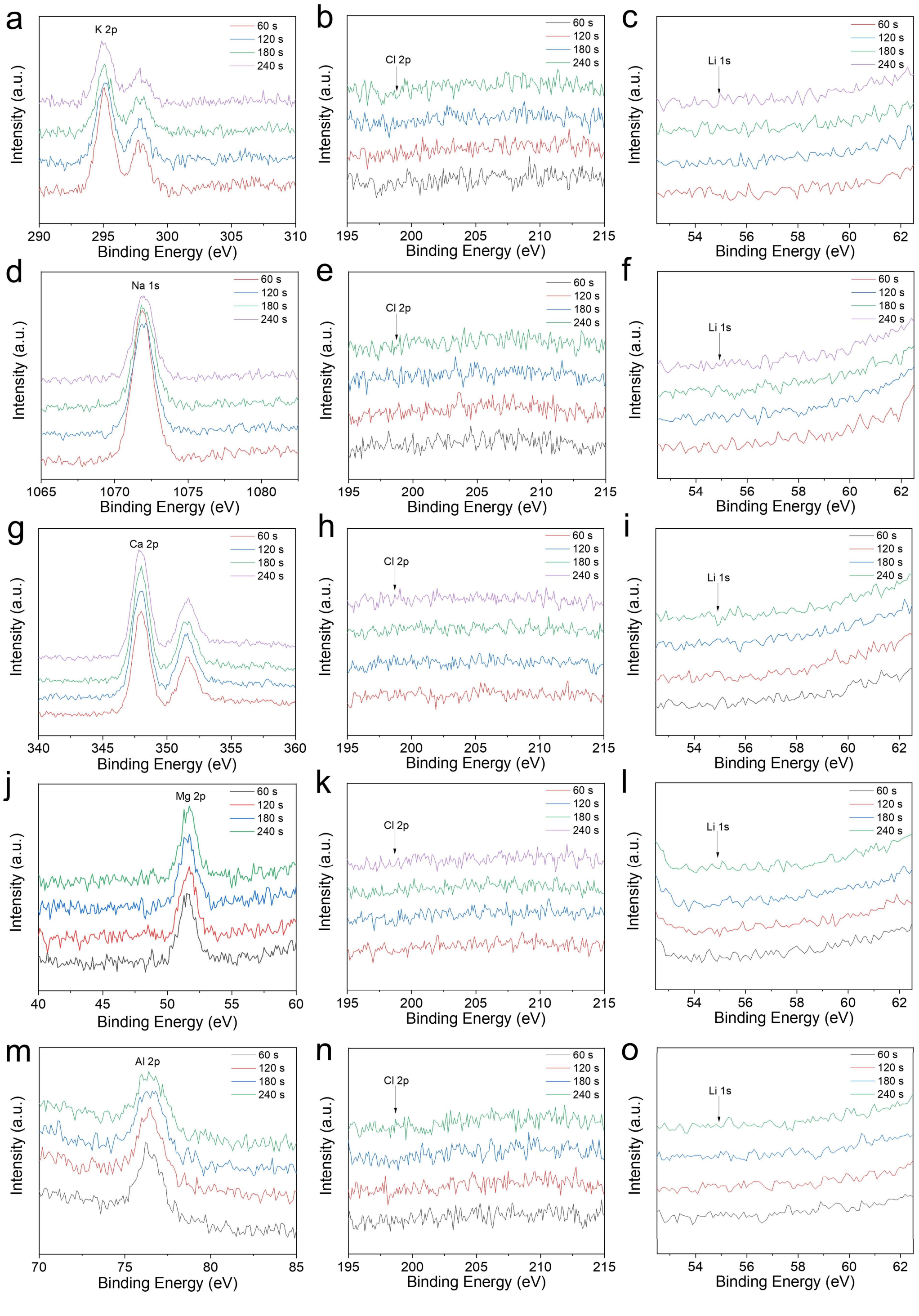

Supplementary Figure 11 | XPS with depth analysis of $CdPS_3$-Z membranes. a-c, K 2*p* (**a**), Cl 2*p* (**b**), Li 1*s* (**c**), **d-f**, Na 1*s* (**d**), Cl 2*p* (**e**), Li 1*s* (**f**), **g-i**, Ca 2*p* (**g**), Cl 2*p* (**h**), Li 1*s* (**i**), **j-l**, Mg 2*p* (**j**), Cl 2*p* (**k**), Li 1*s* (**l**), and **m-o**, Al 2*p* (**m**), Cl 2*p* (**n**), Li 1*s* (**o**) spectra obtained after different etching time for $CdPS_3$-K, $CdPS_3$-Na, $CdPS_3$-Ca, $CdPS_3$-Mg, $CdPS_3$-Al membranes, respectively. No chlorine and lithium were detected.

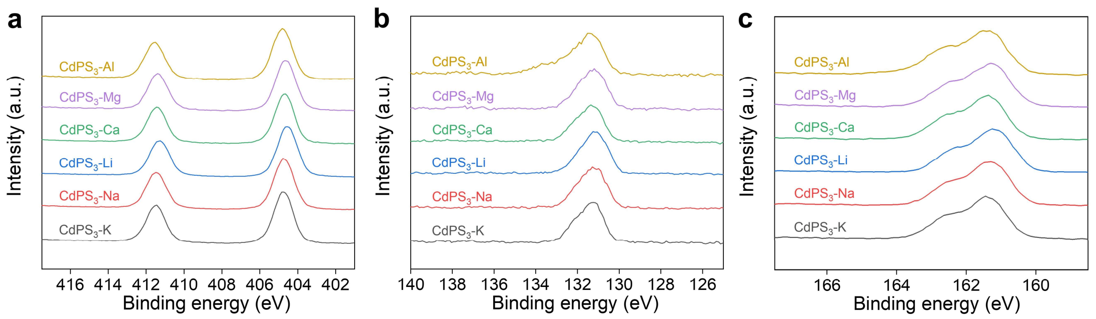

Supplementary Figure 12 | XPS spectra of $CdPS_3$-Z membranes. a-c, Cd 3*d* (**a**), P 2*p* (**b**), and S 2*p* (**c**) XPS spectra of $CdPS_3$-K, $CdPS_3$-Na, $CdPS_3$-Li, $CdPS_3$-Ca, $CdPS_3$-Mg and $CdPS_3$-Al membranes, which show that the valence states of Cd, P and S (+2, +4 and -2, respectively) remain the same in all the membranes.

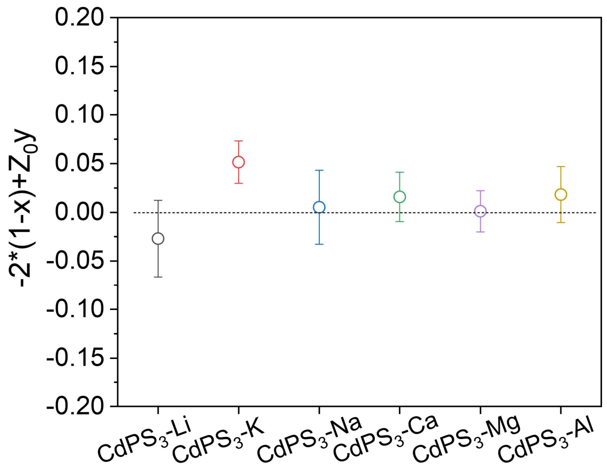

Supplementary Figure 13 | Charge balance in $CdPS_3$-Z membranes. Total charges of $CdPS_3$-Z ($Cd_xPS_3Z_y$) membranes, showing that they keep charge balance. The detailed compositions of membranes were presented in Supplementary Table 1. Z_0 represents the valence state of Z cation.

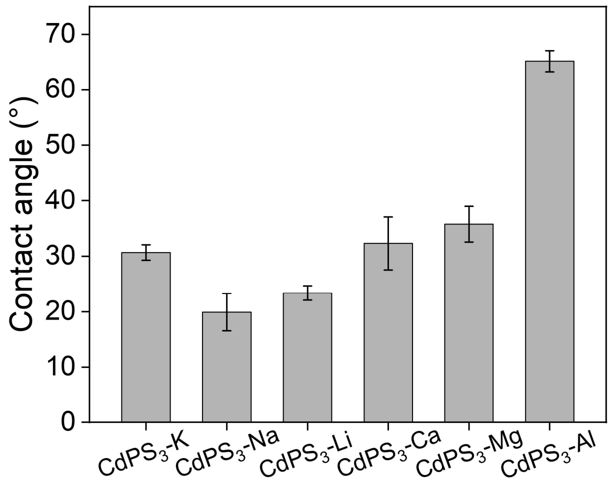

Supplementary Figure 14 | Contact angles toward water for $CdPS_3$-Z membranes that were kept under ambient conditions (~30 – 50% RH).

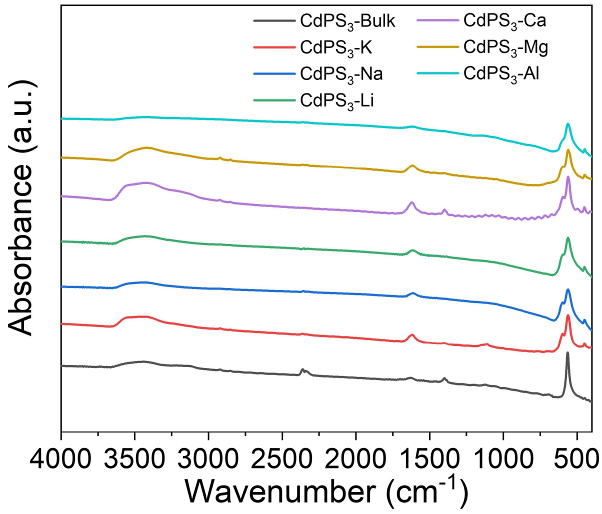

Supplementary Figure 15 | FTIR spectra of bulk $CdPS_3$ crystals and $CdPS_3$-Z membranes at ambient conditions. The peaks at around 3400 cm^{-1} and 1626 cm^{-1} correspond to the ν_{OH} stretching band and δ_{OH} bending band of adsorbed water molecules.

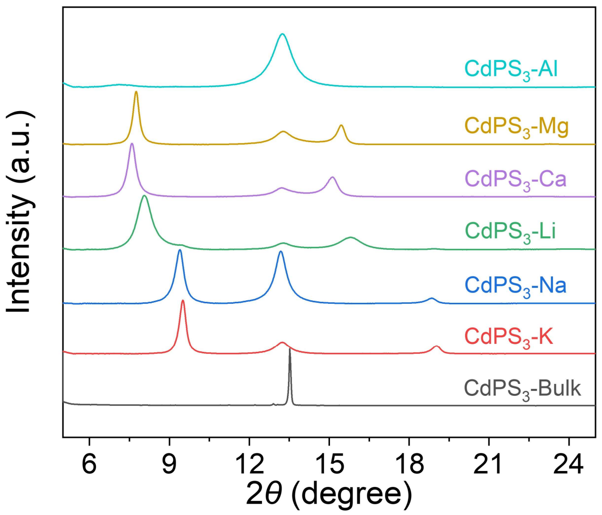

Supplementary Figure 16 | XRD patterns of $CdPS_3$ crystals and $CdPS_3$-Z membranes at ambient conditions. The peaks above 15° is the second-order diffraction of the first peak.

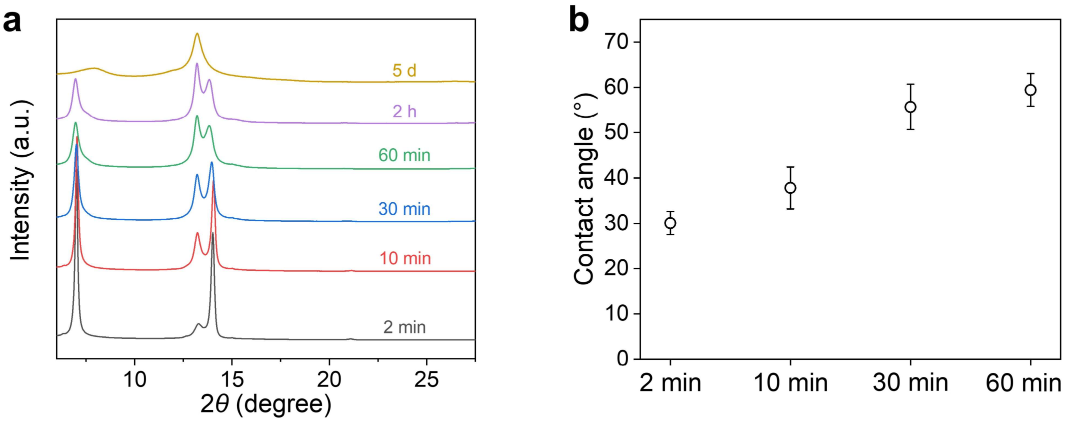

Supplementary Figure 17 | Evolution of XRD patterns (a) and variation of contact angle (b) of $CdPS_3$-Al membrane with drying time under ambient conditions.

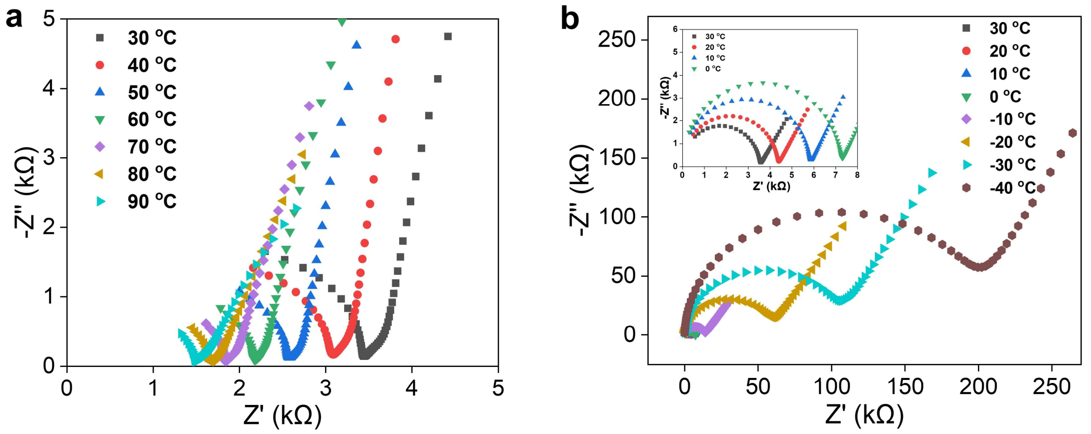

Supplementary Figure 18 | Nyquist plots of $CdPS_3$-K membranes. a, **b**, Nyquist plots of $CdPS_3$-K membranes measured in the temperature range of 30 – 90 °C (**a**) and -40 – 30 °C at 98% RH (**b**).

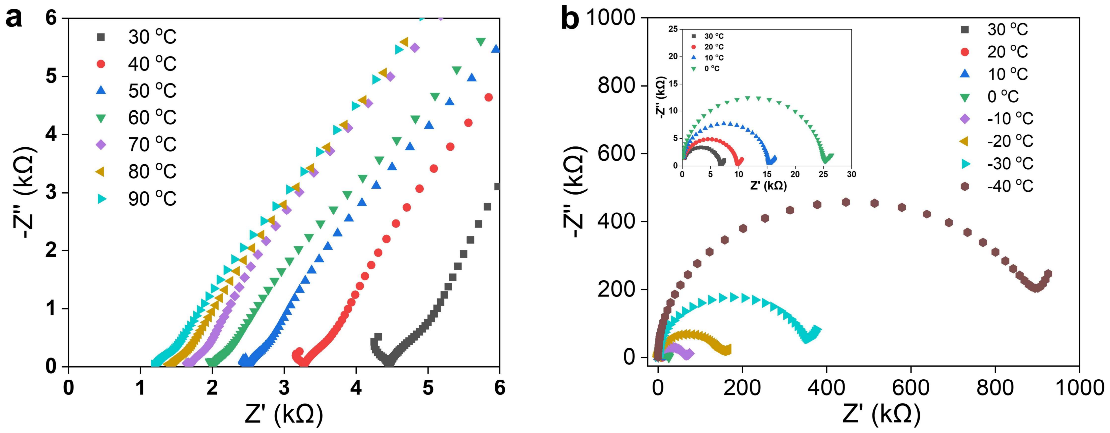

Supplementary Figure 19 | Nyquist plots of $CdPS_3$-Na membranes. a, **b**, Nyquist plots of $CdPS_3$-Na membrane measured in the temperature range of 30 – 90 °C (**a**) and -40 – 30 °C at 98% RH (**b**).

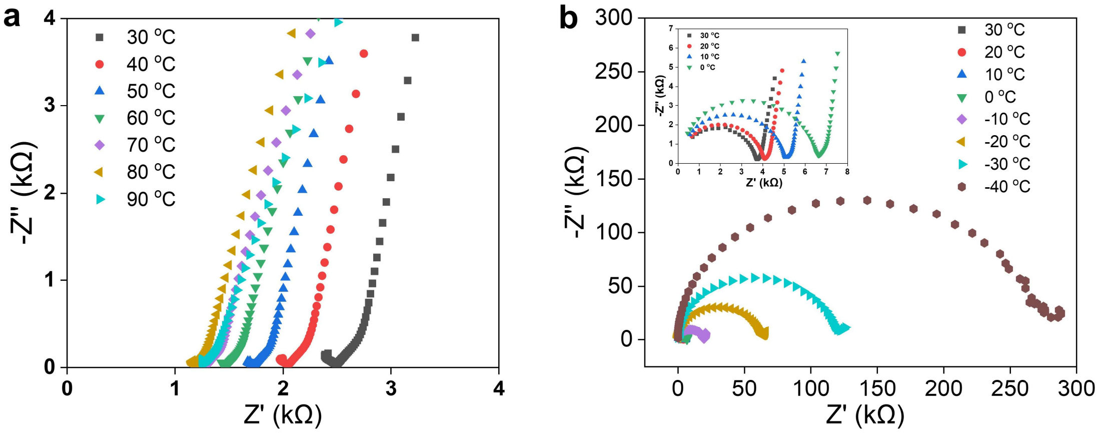

Supplementary Figure 20 | Nyquist plots of $CdPS_3$-Li membranes. a, **b**, Nyquist plots of $CdPS_3$-Li membrane measured in the temperature range of 30 – 90 °C (**a**) and -40 – 30 °C at 98% RH (**b**).

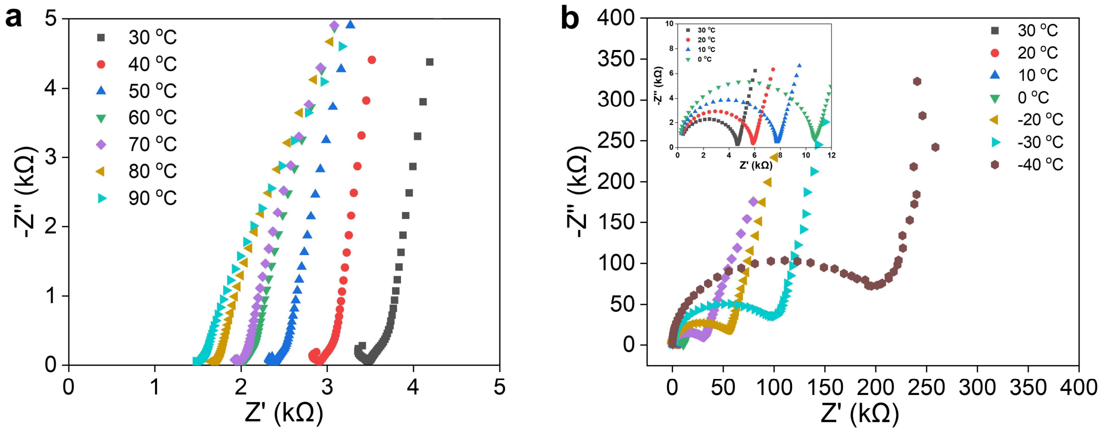

Supplementary Figure 21 | Nyquist plots of $CdPS_3$-Ca membranes. a, **b**, Nyquist plots of $CdPS_3$-Ca membrane measured in the temperature range of 30 – 90 °C (**a**) and -40 – 30 °C at 98% RH (**b**).

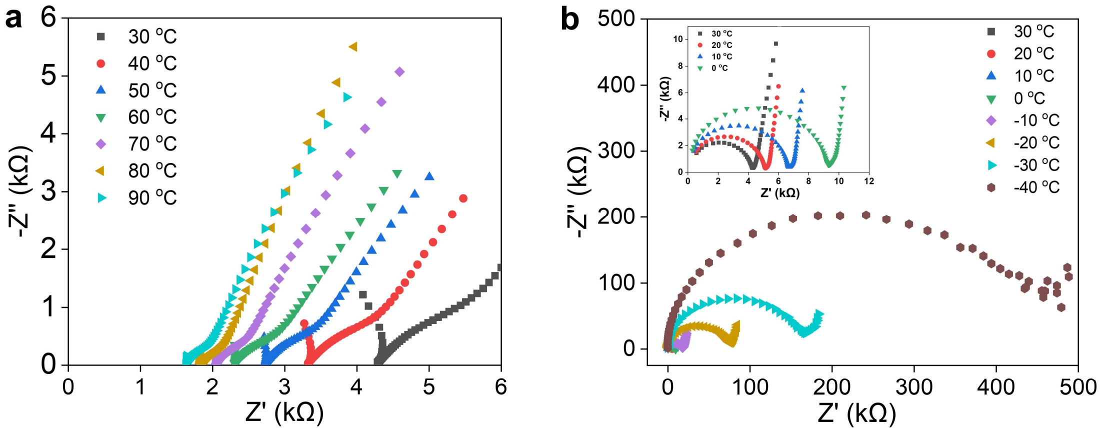

Supplementary Figure 22 | Nyquist plots of $CdPS_3$-Mg membranes. a, **b**, Nyquist plots of $CdPS_3$-Mg membrane measured in the temperature range of 30 – 90 °C (**a**) and -40 – 30 °C at 98% RH (**b**).

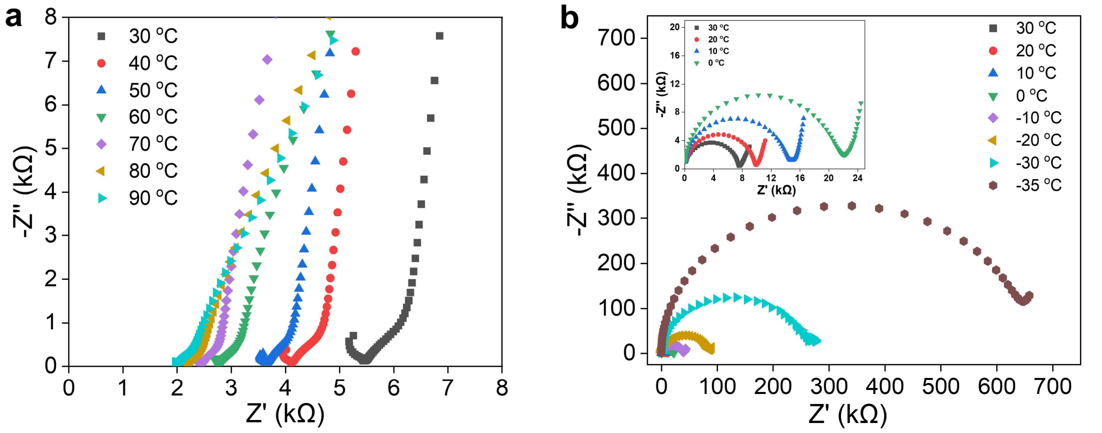

Supplementary Figure 23 | Nyquist plots of $CdPS_3$-Al membranes. a, **b**, Nyquist plots of $CdPS_3$-Al membrane measured in the temperature range of 30 – 90 °C (**a**) and -35 – 30 °C at 98% RH (**b**). The data at -40 °C was not included since the resistance exceeded the detection limit.

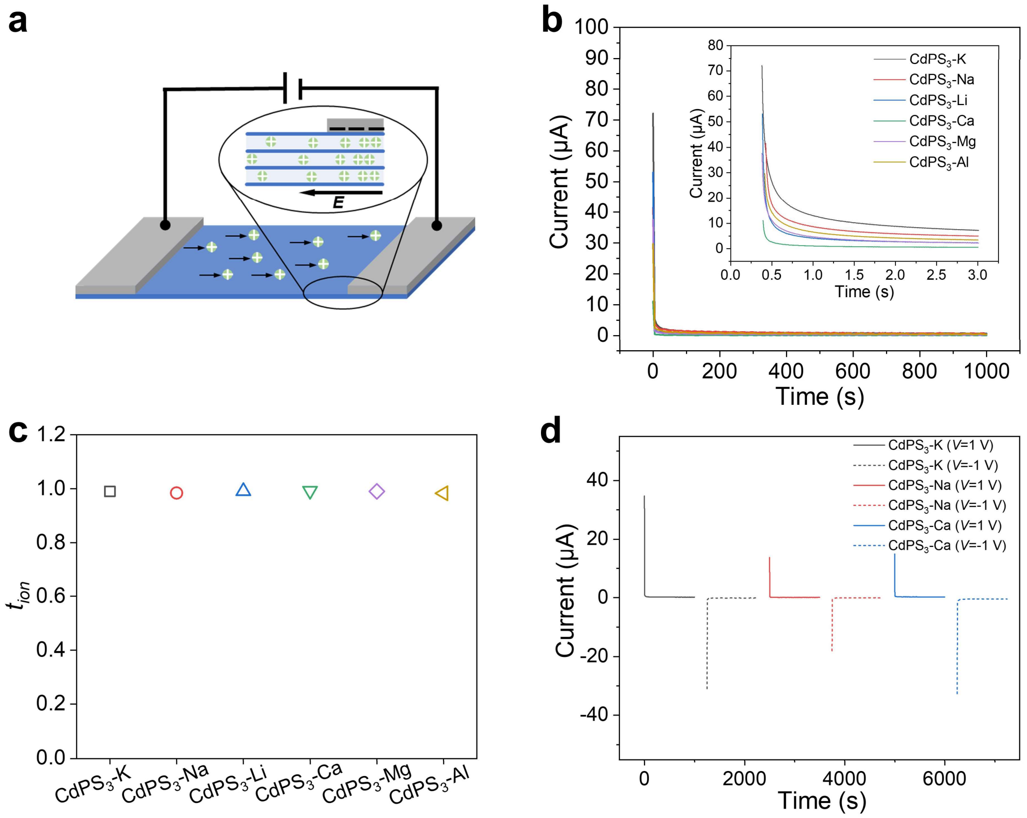

Supplementary Figure 24 | Estimation of the ionic transference numbers of $CdPS_3$-Z membranes. a, Schematic illustration of chronoamperometry method with inert electrodes employed. **b**, Polarization plots measured by chronoamperometry (V = 1 V) at 30 °C and 98% RH. Inset, zoom-in view of polarization plots at the beginning. **c**, The extracted t_{ion} for $CdPS_3$-Z membranes. **d**, Polarization plots at different voltage directions at 30 °C and 98% RH.

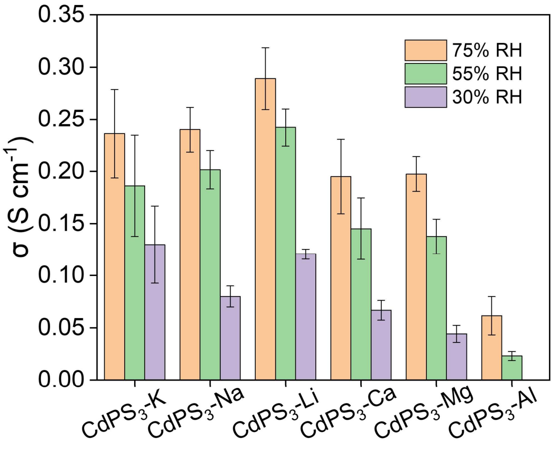

Supplementary Figure 25 | The ion conductivities of $CdPS_3$-Z membranes at low RHs at 60 °C. Three humidity conditions of 75%, 55%, and 30% RH were measured. The ion conductance of $CdPS_3$-Al membrane at 30% RH and 60 °C was below the detection limit of the device and thus, was not included in the graph.

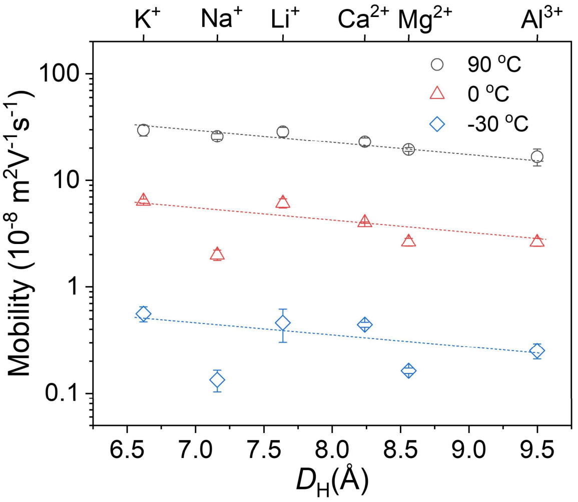

Supplementary Figure 26 | Ion mobilities in $CdPS_3$-Z nanochannels at different temperatures. Ion mobilities in $CdPS_3$-Z nanochannels as a function of D_H of cations at 90, 0, and -30 °C. Dashed lines are guides for eye.

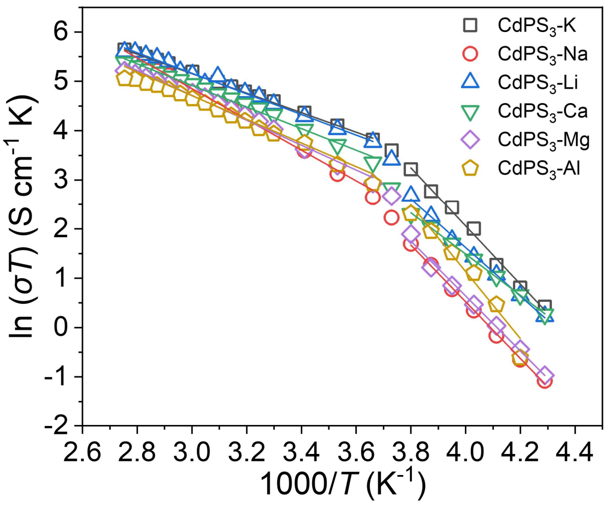

Supplementary Figure 27 | Determinations of the activation energy barriers. Temperature-dependent conductivities of $CdPS_3$-Z membranes at 98% RH with linear fitting according to the Arrhenius equation, the slope of which represents E_a.

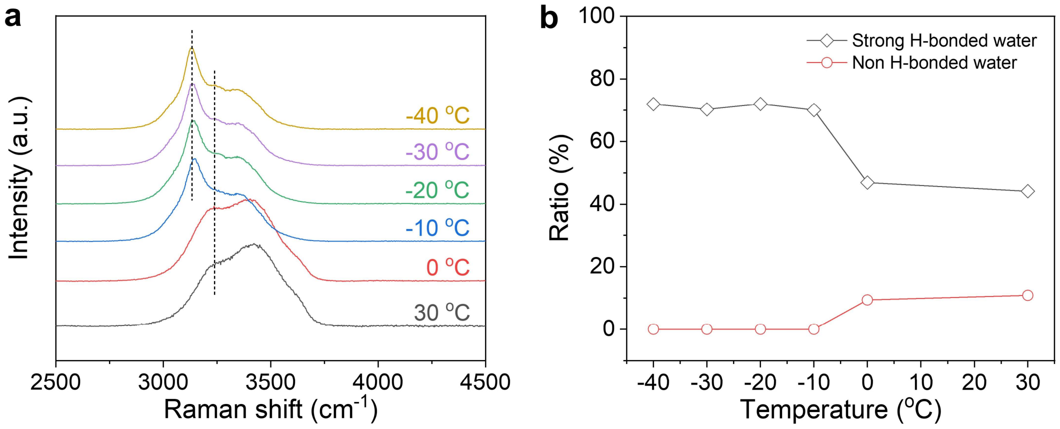

Supplementary Figure 28 | Raman spectra of pure water at different temperatures. **a**, Raman O-H stretching bands. **b**, The corresponding proportion of strongly H-bonded water and non-H-bonded water.

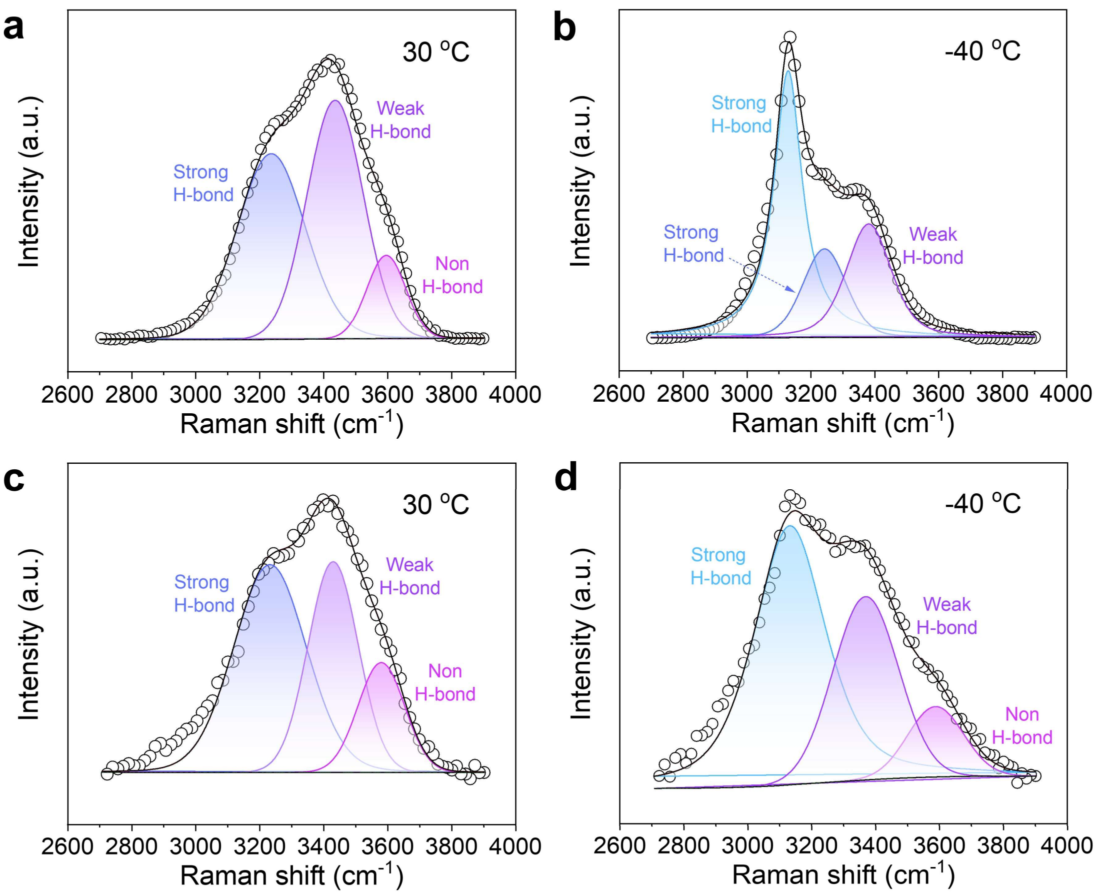

Supplementary Figure 29 | Characterizations on the structure of water molecules by Raman fitting. The fitted O-H stretching bands, showing water molecules with strong, weak and non H-bonds. **a**, **b**, Pure water at 30 °C (**a**) and -40 °C (**b**). **c**, **d**, $CdPS_3$-Li membrane at 30 °C (**c**) and -40 °C (**d**).

Supplementary Tables

Supplementary Table 1. Chemical compositions of $CdPS_3$-Z membranes. The content of Cd and Z in each membrane was determined by ICP-AES. Cl was detected by EDS and XPS, showing no signals within the detection limit.

Name		x	x error	y	y error	Cl	Chemical formula
$CdPS_3$-Li	$Cd_xPS_3Li_y$	0.84	0.01	0.29	0.01	-	$Cd_{0.84}PS_3Li_{0.29}$
$CdPS_3$-K	$Cd_xPS_3K_y$	0.86	0.01	0.33	0.01	-	$Cd_{0.86}PS_3K_{0.33}$
$CdPS_3$-Na	$Cd_xPS_3Na_y$	0.85	0.01	0.31	0.02	-	$Cd_{0.85}PS_3Na_{0.31}$
$CdPS_3$-Ca	$Cd_xPS_3Ca_y$	0.85	0.01	0.15	0.01	-	$Cd_{0.85}PS_3Ca_{0.15}$
$CdPS_3$-Mg	$Cd_xPS_3Mg_y$	0.84	0.01	0.16	0.01	-	$Cd_{0.84}PS_3Mg_{0.16}$
$CdPS_3$-Al	$Cd_xPS_3Al_y$	0.85	0.01	0.11	0.01	-	$Cd_{0.85}PS_3Al_{0.11}$

Supplementary Table 2. K^+ conductivities of various materials.

Type	Material	T (℃)	Conductivity (mS cm^{-1})	Ref.
Liquid ion conductors	Water in salt KCF_3SO_3 (22 m)	25	76	1
		-20	10	
	Organic electrolyte KPF_6/AN	-10~60	27.0~45.5*	2
	Organic electrolyte KPF_6/EC-DMC (1:1)	-10~60	5.0~17.3*	
	Ionic liquid electrolyte K[FSA]-[C_3C_1pyrr][FSA]	-15~120	0.6~38.8	3
Gel ion conductors	Hydrogel KCl/PVA	25	37	4
	Gel polymer KI/PAN	30~100	2×10^{-2}~0.26*	5
Solid ion conductors	$K_6[Rh_4Zn_4O$ (L-cysteinate)$_{12}]$ nH_2O	-83~67	3.8×10^{-5}~17	6
	$K_2Mg_2TeO_6$	25~300	1×10^{-2}~40	7
	K-β''-Al_2O_3	25~400	0.8~108.7*	8
	KSi_2P_3	-20~25	7×10^{-2}~0.27*	9
	Polymer electrolyte KBr/PVC-PEO	30~90	2.5×10^{-2}~4.7*	10
solid ion conductors	$CdPS_3$-K	-40~90	6.5~775.1	This work

Supplementary Table 3. Na^+ conductivities of various materials.

Type	Material	T (℃)	Conductivity (mS cm^{-1})	Ref.
Liquid ion conductors	Aqueous solution Na_2SO_4 (1 M)	0~30	0.02~115*	11
	Water in salt NaOTF (9.26 m)	-10~60	12.7~159.7*	12
	Organic electrolyte $NaClO_4$ (1 M) in EC-DMC	-20~40	1.6~5.9*	13
	Ionic liquid electrolyte $Na_{0.075}BMIm_{0.925}TFSI$	0~60	0.8~10.0*	14
Gel ion conductors	Hydrogel Na_2SO_4 (1 M)/SiO_2 with methyl alcohol as anti-freezing additive	-30~30	0.07~24.7*	11
	Gel electrolyte $NaNTf_2$ (0.1 M)/$C_4mpyrNTf_2$/PMMA	25~100	1.1~9.7*	15
Solid ion conductors	β-alumina (Na_2O-11Al_2O_3)	20-700	20.1~286.4*	16
	94Na_3PS_4-6Na_4SiS_4	25~110	0.8~6.7*	17
	Na_3SbSe_4	25~90	3.7~12.9*	18
	$Na_{3.4}Sc_{0.4}Zr_{1.6}(SiO_4)_2(PO_4)$	25~300	4.0~201.1*	19
	$Na_{11}Sn_2PS_{12}$	-20~50	0.2~2.0*	20
	$Na_2(BH_4)(NH_2)$	20~150	1.3×10^{-3}~2.4*	21
	Polymer electrolyte NaTFSI/$(PEO)_9$	0~70	2×10^{-4}~0.3*	22
	Hybrid electrolyte NASICON/PVdF-HFP/$NaCF_3SO_3$ (TEGDME)	-30~90	4.2×10^{-2}~1.4*	23
	MOF MIT-20-Na	25~70	1.8×10^{-2}~0.11*	24
solid ion conductors	$CdPS_3$-Na	-40~90	1.5~676.4	This work

Supplementary Table 4. Li^+ conductivities of various materials.

Type	Material	T (℃)	Conductivity (mS cm^{-1})	Ref.
Liquid ion conductors	Aqueous electrolyte Li_2SO_4 (1 M)	-20~0	1.2~30.2*	25
	Water/Sulfolane Hybrid Electrolyte (12SL-4H_2O-3$LiClO_4$)	-50~30	0.05~3.05	26
	Organic electrolyte 1M $LiPF_6$/EC-PC (1:1 vol)	-80~25	2×10^{-5}~7.3*	27
	Ternary electrolyte EO/$LiBF_4$/DEMEBF$_4$ (8:1:2)	15~55	0.5~3.1*	28
Gel ion conductors	Gel electrolyte $LiPF_6$ (1 M)/EC-DMC/PVDF-HFP	-5~55	0.9~3.2*	29
Solid ion conductors	LiI-alumina composites	25~300	0.02~15.42*	30
	Glass-ceramic 70Li_2S-30P_2S_5	25~250	2.6~50.1*	31
	$Li_{0.22}La_{0.60}TiO_3$	-20~70	4.3×10^{-2}~4.0*	32
	$Li_{1.2}Ti_{1.8}Al_{0.2}(PO_4)_3$	-100~200	1.6×10^{-3}~92.2*	33
	Ga-Substituted $La_3Zr_2Li_7O_{12}$ garnet	0~86	0.5~6.6*	34
	$Li_{10}GeP_2S_{12}$	-100~100	1×10^{-2}~83.9*	35
	$Li_{9.54}Si_{1.74}P_{1.44}S_{11.7}Cl_{0.3}$	-30~25	3.1~25.5*	36
	$Li_2(BH_4)(NH_2)$	25~105	0.2~60	37
	Polymer electrolyte $LiClO_4$/PEO with 1 wt% TiO_2 nanorods	20~100	1.7×10^{-2}~2.7*	38
	MOF MIT-20-Li	25~70	4.3×10^{-2}~0.17*	24
	BN/IL	-20~30	0.23~4.60*	39
solid ion conductors	$CdPS_3$-Li	-40~90	5.4~747.4	This work

Supplementary Table 5. Ca^{2+} conductivities of various materials.

Type	Material	T (℃)	Conductivity ($mS\ cm^{-1}$)	Ref.
Liquid ion conductors	Organic electrolyte $Ca(TFSI)_2$ (1 M)/$EC_{0.5}$-$PC_{0.5}$	-10~120	3.4~20*	40
Gel ion conductors	Gel electrolyte $Ca(ClO_4)_2$/EMITf/PEGDA	25~110	0.17~2.21	41
	Aqueous gel PVA/$Ca(NO_3)_2$	25	11.56	42
	Gel polymer PEGDA/EC-PC/$Ca(ClO_4)_2$	25~110	4.3×10^{-2}~0.34*	43
Solid ion conductors	Ca-β''-alumina	60~400	1.9×10^{-2}~84.6*	44
	Polymer electrolyte $Ca(NO_3)_2$/PTHF-Epoxy	30~110	0.1~24.0*	45
	Polymer electrolyte $Ca(CF_3SO_3)_2$/PEO	20~90	4×10^{-4}~0.5*	46
solid ion conductors	$CdPS_3$-Ca	-40~90	5.6~601.1	This work

Supplementary Table 6. Mg^{2+} conductivities of various materials.

Type	Material	T (℃)	Conductivity (mS cm^{-1})	Ref.
Liquid ion conductors	Organic electrolyte $Mg(ClO_4)_2$ (1 M)/$EC_{0.5}$-$PC_{0.5}$	-10~100	1.2~12.6	40
	Organic electrolyte $Mg(PF_6)_2(CH_3CN)_6$/THF-CH_3CN	25	28.3	47
Gel ion conductors	Gel polymer $Mg(ClO_4)_2$/PVdF-HFP/MgO	0~80	2~10	48
Solid ion conductors	$Mg(NH_2CH_2CH_2NH_2)(BH_4)_2$	30~70	5×10^{-5}~6×10^{-2}	49
	$MgSc_2Se_4$	25~80	0.4~1.1*	50
	Polymer electrolyte $Mg(ClO_4)_2/PEO_{12}$	20~120	3×10^{-4}~0.34*	51
	Polymer electrolyte $Mg(ClO_4)_2$/PVA-PAN	30~70	0.3~0.7*	52
	Polymer electrolyte $Mg(BH_4)_2$-PTHF/glass fiber	0~100	0.1~4.0*	53
	Mg_2(dobdc)-$Mg(TFSI)_2$ under MeOH vapor	20~35	0.23~0.38*	54
	MOF MIT-20-Mg	25~70	8.6×10^{-4}~5.1×10^{-3}*	24
solid ion conductors	$CdPS_3$-Mg	-40~90	1.6~506.0	This work

Supplementary Table 7. Al^{3+} conductivities of various materials.

Type	Material	T (℃)	Conductivity ($mS\ cm^{-1}$)	Ref.
Liquid ion conductors	Water-in-salt $Al(OTF)_3$ (5 m)	25~60	25.9~64.2*	55
	Molten mixtures $AlCl_3$/DMTC	-31~123	4.02×10^{-2}~77.8	56
	Ionic liquid electrolyte [BMIM]OTF/$Al(OTF)_3$ (0.05 M)	0~80	0.8~14.3*	57
Gel ion conductors	Gel polymer EMImCl-AlCl3/polyacrylamide	-20~100	0.2~10.7*	58
Solid ion conductors	Polymer electrolyte $Al(NO_3)_3$/PTHF-Epoxy	20~110	2.2×10^{-4}~2.9×10^{-2}*	59
	Hybrid polymer electrolyte $AlCl_3$-Emim FSI/PEO/SiO_2	25~100	0.68~5.84*	60
	Polymer electrolyte $Al(Tf)_3$-EMITf/PVdF-HFP	30-90	1.7~5.6*	61
solid ion conductors	$CdPS_3$-Al	-35~90	2.3~432.8	This work

*The values in Supplementary Table 2 to 7 were calculated based on the data extracted from the figures in the literatures.

Supplementary References

1. Jiang, L. W. et al. Building aqueous K-ion batteries for energy storage. *Nat. Energy* **4**, 495-503 (2019).
2. Amara, S. et al. Comparative study of alkali-cation-based (Li^+, Na^+, K^+) electrolytes in acetonitrile and alkylcarbonates. *ChemPhysChem* **20**, 581-594 (2019).
3. Yamamoto, T., Matsumoto, K., Hagiwara, R. & Nohira, T. Physicochemical and electrochemical properties of $K[N(SO_2F)_2]$–[*N*-Methyl-*N*-propylpyrrolidinium]$[N(SO_2F)_2]$ ionic liquids for potassium-ion batteries. *J. Phys. Chem. C* **121**, 18450-18458 (2017).
4. Lu, K., Zhang, H., Gao, S. Y., Cheng, Y. W. & Ma, H. Y. High rate and stable symmetric potassium ion batteries fabricated with flexible electrodes and solid-state electrolytes. *Nanoscale* **10**, 20754-20760 (2018).
5. Jyothi, N. K., Venkataratnam, K. K., Murty, P. N. & Kumar, K. V. Preparation and characterization of PAN–KI complexed gel polymer electrolytes for solid-state battery applications. *Bull. Mater. Sci.* **39**, 1047-1055 (2016).
6. Yoshinari, N., Yamashita, S., Fukuda, Y., Nakazawa, Y. & Konno, T. Mobility of hydrated alkali metal ions in metallosupramolecular ionic crystals. *Chem. Sci.* **10**, 587-593 (2019).
7. Masese, T. et al. Rechargeable potassium-ion batteries with honeycomb-layered tellurates as high voltage cathodes and fast potassium-ion conductors. *Nat. Commun.* **9**, 3823 (2018).

8. Lu, X. C., Bowden, M. E., Sprenkle, V. L. & Liu, J. A low cost, high energy density, and long cycle life potassium-sulfur battery for grid-scale energy storage. *Adv. Mater.* **27**, 5915-5922 (2015).

9. Haffner, A. et al. Polymorphism and fast potassium-ion conduction in the T5 supertetrahedral phosphidosilicate KSi_2P_3. *Angew. Chem.-Int. Edit.* **60**, 13641-13646 (2021).

10. Nadimicherla, R., Sharma, A. K., Rao, V. V. R. N. & Chen, W. Electrical and solid-state battery performance of a new PVC/PEO + KBr blend-based polymer electrolyte system. *Ionics* **21**, 1587-1594 (2014).

11. Cheng, Y. B., Chi, X. W., Yang, J. H. & Liu, Y. Cost attractive hydrogel electrolyte for low temperature aqueous sodium ion batteries. *J. Energy Storage* **40**, 102701 (2021).

12. Suo, L. M. et al. "Water-in-salt" electrolyte makes aqueous sodium-ion battery safe, green, and long-lasting. *Adv. Energy Mater.* **7**, 1701189 (2017).

13. Bhide, A., Hofmann, J., Durr, A. K., Janek, J. & Adelhelm, P. Electrochemical stability of non-aqueous electrolytes for sodium-ion batteries and their compatibility with $Na_{0.7}CoO_2$. *Phys. Chem. Chem. Phys.* **16**, 1987-1998 (2014).

14. Monti, D., Jonsson, E., Palacin, M. R. & Johansson, P. Ionic liquid based electrolytes for sodium-ion batteries: Na^+ solvation and ionic conductivity. *J. Power Sources* **245**, 630-636 (2014).

15. Noor, S. A. M., Yoon, H., Forsyth, M. & MacFarlane, D. R. Gelled ionic liquid sodium ion conductors for sodium batteries. *Electrochim. Acta* **169**, 376-381

(2015).

16. Adachi, G.-Y., Imanaka, N. & Aono, H. Fast Li⊕ conducting ceramic electrolytes. *Adv. Mater.* **8**, 127-135 (1996).

17. Tanibata, N., Noi, K., Hayashi, A. & Tatsumisago, M. Preparation and characterization of highly sodium ion conducting Na_3PS_4–Na_4SiS_4 solid electrolytes. *RSC Adv.* **4**, 17120-17123 (2014).

18. Wang, N. et al. Improvement in ion transport in Na_3PSe_4–Na_3SbSe_4 by Sb substitution. *J. Mater. Sci.* **53**, 1987-1994 (2017).

19. Ma, Q. L. et al. Scandium-substituted $Na_3Zr_2(SiO_4)_2(PO_4)$ prepared by a solution assisted solid-state reaction method as sodium-ion conductors. *Chem. Mater.* **28**, 4821-4828 (2016).

20. Zhang, Z. et al. $Na_{11}Sn_2PS_{12}$: a new solid state sodium superionic conductor. *Energy Environ. Sci.* **11**, 87-93 (2018).

21. Matsuo, M. et al. Sodium and magnesium ionic conduction in complex hydrides. *J. Alloy Compd.* **580**, S98-S101 (2013).

22. Boschin, A. & Johansson, P. Characterization of NaX (X: TFSI, FSI) – PEO based solid polymer electrolytes for sodium batteries. *Electrochim. Acta* **175**, 124-133 (2015).

23. Kim, J. K., Lim, Y. J., Kim, H., Cho, G. B. & Kim, Y. A hybrid solid electrolyte for flexible solid-state sodium batteries. *Energy Environ. Sci.* **8**, 3589-3596 (2015).

24. Park, S. S., Tulchinsky, Y. & Dinca, M. Single-ion Li^+, Na^+, and Mg^{2+} solid electrolytes supported by a mesoporous anionic Cu-azolate metal-organic

framework. *J. Am. Chem. Soc.* **139**, 13260-13263 (2017).

25. Tron, A., Jeong, S., Park, Y. D. & Mun, J. Aqueous lithium-ion battery of nano-$LiFePO_4$ with antifreezing agent of ethyleneglycol for low-temperature operation. *ACS Sustainable Chem. Eng.* **7**, 14531-14538 (2019).
26. Liu, J. H. et al. Water/sulfolane hybrid electrolyte achieves ultralow-temperature operation for high-voltage aqueous lithium-ion batteries. *Adv. Funct. Mater.* **32**, 2106811 (2021).
27. Stallworth, P. E. et al. NMR, DSC and high pressure electrical conductivity studies of liquid and hybrid electrolytes. *J. Power Sources* **81**, 739-747 (1999).
28. Egashira, M., Asai, T., Yoshimoto, N. & Morita, M. Ionic conductivity of ternary electrolyte containing sodium salt and ionic liquid. *Electrochim. Acta* **58**, 95-98 (2011).
29. Song, J. Y., Wang, Y. Y. & Wan, C. C. Conductivity study of porous plasticized polymer electrolytes based on poly(vinylidene fluoride) - A comparison with polypropylene separators. *J. Electrochem. Soc.* **147**, 3219-3225 (2000).
30. Poulsen, F. W., Andersen, N. H., Kindl, B. & Schoonman, J. Properties of LiI - alumina composite electrolytes. *Solid State Ionics* **9-10**, 119-122 (1983).
31. Mizuno, F., Hayashi, A., Tadanaga, K. & Tatsumisago, M. New, highly ion-conductive crystals precipitated from Li_2S-P_2S_5 glasses. *Adv. Mater.* **17**, 918-921 (2005).
32. Kwon, W. J. et al. Enhanced Li^+ conduction in perovskite $Li_{3x}La_{2/3-x}\square_{1/3-2x}TiO_3$ solid-electrolytes via microstructural engineering. *J. Mater. Chem. A* **5**, 6257-6262

(2017).

33. Arbi, K., Hoelzel, M., Kuhn, A., Garcia-Alvarado, F. & Sanz, J. Structural factors that enhance lithium mobility in fast-ion $Li_{1+x}Ti_{2-x}Al_x(PO_4)_3$ ($0 \leq x \leq 0.4$) conductors investigated by neutron diffraction in the temperature range 100–500 K. *Inorg. Chem.* **52**, 9290-9296 (2013).

34. Bernuy-Lopez, C. et al. Atmosphere controlled processing of Ga-substituted garnets for high Li-ion conductivity ceramics. *Chem. Mater.* **26**, 3610-3617 (2014).

35. Kamaya, N. et al. A lithium superionic conductor. *Nat. Mater.* **10**, 682-686 (2011).

36. Kato, Y. et al. High-power all-solid-state batteries using sulfide superionic conductors. *Nat. Energy* **1**, 16030 (2016).

37. Matsuo, M. et al. Complex hydrides with $(BH_4)^-$ and $(NH_2)^-$ anions as new lithium fast-ion conductors. *J. Am. Chem. Soc.* **131**, 16389-16391 (2009).

38. Vasudevan, S. & Fullerton-Shirey, S. K. Effect of nanoparticle shape on the electrical and thermal properties of solid polymer electrolytes. *J. Phys. Chem. C* **123**, 10720-10726 (2019).

39. Li, M. T. et al. Graphene-analogues boron nitride nanosheets confining ionic liquids: A high-performance quasi-liquid solid electrolyte. *Small* **12**, 3535-3542 (2016).

40. Tchitchekova, D. S. et al. On the reliability of half-cell tests for monovalent (Li^+, Na^+) and divalent (Mg^{2+}, Ca^{2+}) cation based batteries. *J. Electrochem. Soc.* **164**, A1384-A1392 (2017).

41. Biria, S., Pathreeker, S., Genier, F. S. & Hosein, I. D. A highly conductive and

thermally stable ionic liquid gel electrolyte for calcium-ion batteries. *ACS Appl. Polym. Mater.* **2**, 2111-2118 (2020).

42. Tang, X. et al. A universal strategy towards high-energy aqueous multivalent-ion batteries. *Nat. Commun.* **12**, 2857 (2021).
43. Biria, S. et al. Gel polymer electrolytes based on cross-linked poly(ethylene glycol) diacrylate for calcium-ion conduction. *ACS Omega* **6**, 17095-17102 (2021).
44. Farrington, G. C. & Dunn, B. Divalent beta″-aluminas: High conductivity solid electrolytes for divalent cations. *Solid State Ionics* **7**, 267-281 (1982).
45. Wang, J. Y., Genier, F. S., Li, H. S., Biria, S. & Hosein, I. D. A solid polymer electrolyte from cross-linked polytetrahydrofuran for calcium ion conduction. *ACS Appl. Polym. Mater.* **1**, 1837-1844 (2019).
46. Martinez-Cisneros, C. S. et al. Opening the door to liquid-free polymer electrolytes for calcium batteries. *Electrochim. Acta* **353**, 136525 (2020).
47. Keyzer, E. N. et al. $Mg(PF_6)^{2-}$ based electrolyte systems: Understanding electrolyte-electrode interactions for the development of Mg-ion batteries. *J. Am. Chem. Soc.* **138**, 8682-8685 (2016).
48. Pandey, G. P., Agrawal, R. C. & Hashmi, S. A. Magnesium ion-conducting gel polymer electrolytes dispersed with nanosized magnesium oxide. *J. Power Sources* **190**, 563-572 (2009).
49. Roedern, E., Kuhnel, R. S., Remhof, A. & Battaglia, C. Magnesium ethylenediamine borohydride as solid-state electrolyte for magnesium batteries. *Sci. Rep.* **7**, 46189 (2017).

50. Canepa, P. et al. High magnesium mobility in ternary spinel chalcogenides. *Nat. Commun.* **8**, 1759 (2017).

51. Patrick, A., Glasse, M., Latham, R. & Linford, R. Novel solid-state polymeric batteries. *Solid State Ionics* **18-9**, 1063-1067 (1986).

52. Manjuladevi, R. et al. Mg-ion conducting blend polymer electrolyte based on poly(vinyl alcohol)-poly (acrylonitrile) with magnesium perchlorate. *Solid State Ionics* **308**, 90-100 (2017).

53. Du, A. B. et al. A crosslinked polytetrahydrofuran-borate-based polymer electrolyte enabling wide-working-temperature-range rechargeable magnesium batteries. *Adv. Mater.* **31**, 1805930 (2019).

54. Yoshida, Y., Kato, K. & Sadakiyo, M. Vapor-induced superionic conduction of magnesium ions in a metal–organic framework. *J. Phys. Chem. C* **125**, 21124-21130 (2021).

55. Zhou, A. X. et al. Water-in-salt electrolyte promotes high-capacity $FeFe(CN)_6$ cathode for aqueous Al-ion battery. *ACS Appl. Mater. Interfaces* **11**, 41356-41362 (2019).

56. Vestergaard, B. et al. Molten triazolium chloride systems as new aluminum battery electrolytes. *J. Electrochem. Soc.* **140**, 3108-3113 (1993).

57. Wang, H. L. et al. High-voltage and noncorrosive ionic liquid electrolyte used in rechargeable aluminum battery. *ACS Appl. Mater. Interfaces* **8**, 27444-27448 (2016).

58. Sun, X. G. et al. Polymer gel electrolytes for application in aluminum deposition

and rechargeable aluminum ion batteries. *Chem. Commun.* **52**, 292-295 (2016).

59. Yao, T. Y., Genier, F. S., Biria, S. & Hosein, I. D. A solid polymer electrolyte for aluminum ion conduction. *Results Phys.* **10**, 529-531 (2018).
60. Song, S. F. et al. Al conductive hybrid solid polymer electrolyte. *Solid State Ionics* **300**, 165-168 (2017).
61. Liu, J. H. et al. A study of low-temperature solid-state supercapacitors based on Al-ion conducting polymer electrolyte and graphene electrodes. *J. Power Sources* **488**, 229461 (2021).
